# Quiet Eye and Computerized Precision Tasks in First-Person Shooter Perspective Esport Games

**DOI:** 10.3389/fpsyg.2021.676591

**Published:** 2021-11-08

**Authors:** Mats Dahl, Mårten Tryding, Alexander Heckler, Marcus Nyström

**Affiliations:** ^1^Department of Psychology, Lund University, Lund, Sweden; ^2^Lund University Humanities Lab, Lund, Sweden

**Keywords:** e-sport, quiet eye, eye-tracking, FPS-game, cognitive load

## Abstract

The gaze behavior in sports and other applied settings has been studied for more than 20 years. A common finding is related to the “quiet eye” (QE), predicting that the duration of the last fixation before a critical event is associated with higher performance. Unlike previous studies conducted in applied settings with mobile eye trackers, we investigate the QE in a context similar to esport, in which participants click the mouse to hit targets presented on a computer screen under different levels of cognitive load. Simultaneously, eye and mouse movements were tracked using a high-end remote eye tracker at 300 Hz. Consistent with previous studies, we found that longer QE fixations were associated with higher performance. Increasing the cognitive load delayed the onset of the QE fixation, but had no significant influence on the QE duration. We discuss the implications of our results in the context of how the QE is defined, the quality of the eye-tracker data, and the type of analysis applied to QE data.

## Introduction

The gaze behavior in various kind of sports and the possibility for novices to enhance performance by trying to mimic experts’ gaze behavior has been studied for more than 20 years (for an overview, see Lebeau et al., 2016; Kredel et al., 2017; Hüttermann et al., 2018). This finding is not only limited to sports, however, but is also found in other applied settings such as shooting performance of police officers (Vickers and Lewinski, 2012) and surgical skills of medical personnel (Causer et al., 2014). In this study, we investigate another type of task: a computerized precision task, similar to those found in computerized first-person shooter (FPS) games in which participants aim and shoot at targets, and where accuracy and speed are often essential for success. We also investigate the effects of increased cognitive load on performance. Finally, we investigate and discuss the influence of parameter selection when defining gaze characteristics known as the “quiet eye,” which have been shown to correlate with expert performance in a number of applied sport settings.

### The Quiet Eye

There are several gaze metrics that can be used as indirect measures of attention and visuomotor behavior. The quiet eye (QE) is the name of a gaze behavior often used as a measure to study the relationship between perceptual behavior and proficiency in various sport-related tasks, e.g., shotgun shooting (trap and double trap), golf putting, and basketball free throws (for an overview, see, e.g., Hüttermann et al., 2018). The term was coined by Vickers (1996) and defined as the duration of the final fixation, in a given task, on the object or location prior to a task-critical action. The final fixation was specified as the last fixation within a three-degree angle for a minimum of 100 msec on the object (see Vickers, 2016 for a background and overview of the phenomena). The QE is considered to start when the performer fixates on the relevant object, and initiates a motor response (QE onset), and ends when the gaze deviates from the object by more than three degrees (QE offset). Both the onset and the duration of the QE have been shown to predict the level of performance, with experts exhibiting an earlier onset and a longer duration of QE than less-skillful performers. This has been found in a variety of sports (e.g., shotgun shooting: Causer et al., 2010; basketball: Rienhoff et al., 2013; biathlon shooting: Vickers and Williams, 2007; dart-throwing: Nibbeling et al., 2012; soccer: Piras and Vickers, 2011), but also in other applied activities (e.g., surgical skills: Causer et al., 2014; visuomotor coordination in children with developmental coordination problems: Miles et al., 2015; decision making within law enforcement: Vickers and Lewinski, 2012).

While an abundance of research has shown a typical QE pattern consistent over a large range of various experimental settings, there is no adequate and generally accepted explanation for the biological underpinnings of this distinct gaze pattern (for an overview and discussion of the causal mechanisms, see Wilson et al., 2015; Rienhoff et al., 2016; Vickers, 2016; Gonzalez et al., 2017). Vickers (2009) suggested that QE reflects the time needed to fine-tune a motor program, where a prolonged QE, with an earlier QE onset, is predictive for executing a successful behavioral response (e.g., a golf putt or a basketball free throw). This is related to functions of attentional control. Vickers (2016) proposed an explanation based on the interaction between the dorsal attentional network (DAN) and the ventral attentional network (VAN) (Corbetta and Shulman, 2002; Corbetta et al., 2008). The two networks have different projection routes and can be considered distinct neural structures, but functionally interactive. The VAN system allocates attentional resources to detect unexpected and intrusive stimuli, while the DAN system tries to maintain a task-relevant focus and thus blocks information from the VAN system (Vickers, 2016). Furthermore, Vickers recently also argued that QE was the reason that the “hot hand” exists in sports^1^ (Vickers, 2016). Her interpretation has been questioned, however. Klostermann and Hossner (2018) propose an “inhibition hypothesis,” which suggests that experts develop a specific solution to a problem and other less optimal solutions are suppressed during the prolonged QE duration. Based on the research currently available, it is not possible to convincingly dismiss either of the interpretations, but as shown in a recent review by Klostermann and Moeinirad (2020), the prolonged QE typically found in expert performance is a robust phenomenon.

Furthermore, whether the focus of attention is external or internal has shown to be of importance (Moore et al., 2012; Klostermann et al., 2014). The results by Moore et al. (2012) indicate that an external focus is less disruptive and cognitively demanding than an internal focus, something that promotes a more efficient use of the cognitive processes guiding and adjusting the motor program. Basically, this means that a longer QE, in which the cognitive resources are allocated to task-relevant demands, provides a possibility to process the acquired goal information more efficiently and initiate a well-tuned and successful motor response.

### Quiet-Eye and Cognitive Load

In sports, elite athletes are often under increased levels of anxiety or mental pressure when competing; for instance, when in the lead at the last shot in a biathlon, or when putting for a win in a golf tournament. Several researchers have shown that a successful performance under pressure is characterized by a longer QE duration compared to those who choked or did not perform at their maximum (see Vickers, 2016 for an overview). According to Vickers (2016), high pressure or anxiety divert cognitive resources from relevant tasks or stimuli, making it harder to maintain an efficient QE pattern. As a consequence, the level of performance is negatively affected (for an overview, see Wilson et al., 2015).

A commonly used induction of cognitive load is the Stroop task (Stroop, 1935). In order to investigate the effects of cognitive load, we used a reverse-Stroop task (Wood et al., 2016) in which the task was to ignore the color that a word was written in, and instead respond to what color the word spelled out. Cognitive load can be introduced in several ways (reduced time frame, ill defined task, increased level of difficulty, etc.). We use the Stroop-task since it is a commonly used and reliable method to increase the cognitive load, but we acknowledge at the same time that there are other forms of cognitive load not covered by the Stroop-manipulation.

### Methodological Issues With Defining and Computing Quiet Eye

A fundamental question concerns the definition of QE; the traditionally used definition (Vickers, 1996) is a fixation for at least 100 msec, within an angle of three degrees of the target. This definition is probably linked to the technical capacity of mobile eye trackers at the time (normally a sampling rate of 50–60 Hz, and an accuracy of one degree at best), and the commonly used threshold of 100 ms as a minimum fixation duration. In this study, on the other hand, a high-resolution remote eye tracker (300 Hz sampling rate and accuracy typically < 1 degree) is used. This raises the question of whether Vickers’s threshold of three degrees could be lowered, and how this would influence the QE. Furthermore, according to Vickers (1996), the QE period should dichotomously discriminate between “hits” and “misses,” That is, a “hit” is when, i.e., a shot is within a designated target area, and a “miss” when it is outside that area. Williams (2016), however, argues that the reason why the QE period should discriminate dichotomously between hits and misses is not obvious. In many settings, there are no distinct borders clearly defining a “hit” area. Rather, the designated “hit area” comprises different sub-areas: the center area is a perfect hit, the area outside the center is close-to-perfect, and so on. This is typically the case for target areas seen in, e.g., trap shooting or archery. Accordingly, a more elaborate way could be to analyze performance as a continuous variable, i.e., to test if the gaze patterns related to QE correlate with the distance between the “bullseye” and the actual “hit.”

The high-resolution eye trackers also allow us to analyze small eye movements dividing long fixations that may pass unnoticed with less-sensitive eye trackers. As Gonzalez et al. (2017) point out, the eye is seldom “quiet” and during the fixation of an eye, there are low-velocity drifts as well as high-velocity microsaccades. To what extent this affects not only our understanding of QE, but also of physical performance, remains unclear (for an overview of fixational eye movements, see, e.g., Collewijn and Kowler, 2008).

### Esport and Quiet Eye

Esport is relatively new, but growing rapidly. Hamari and Sjöblom (2017) describe it as “a form of sports where the primary aspects of the sports are facilitated by electronic systems; the input of players and teams as well as the output of the Esports system are mediated by human–computer interfaces” (p. 211). Parallel to the gaming industry, the number of studies investigating the impact of gaming on different cognitive functions is rapidly increasing (for overview see Dale et al., 2020; Reitman et al., 2020). Many of the popular action video games (AVGs) have a first-person shooter perspective (i.e., *Counter Strike*, *Call of Duty*, *Doom*). AVGs often require focused attention and quick information processing in order to execute very precise and swift movements with the computer mouse combined with clicks, “shots,” to hit targets on the screen. When comparing non-gamers with experienced gamers the latter typically show increased proficiency in a number of processing skills. Several studies using a meta-analytic approach (Powers et al., 2013; Wang et al., 2016; Bediou et al., 2018), have shown robust positive effects of AVG training on several cognitive functions. For instance, experienced gamers react faster, with a maintained accuracy level or hit rate (Gorbet and Sergio, 2018; Pardina-Torner et al., 2019). They also exhibit higher hitrate and lower false alarm rate than less experienced gamers, indicating a better ability to accurately ignore interferences, they perform better on perceptual discrimination tasks where the task is to identify small or low-contrast stimuli (Li et al., 2010). Some studies have also used eye trackers to investigate gaze patterns. For instance, Choi and Kim (2015) found a difference in general eye-movement patterns between novices and experts. This was also shown by Koposov et al. (2020), who found that experts responded more quickly to visual stimuli than novices. However, to our knowledge, there are no previous studies in which QE has been measured in relation to esport.

According to Vickers (2016), QE should be, if possible, measured *in situ*, something that imposes practical problems in many settings. The data become noisy or flawed due to artifacts such as rapid head movements and system inertia. Furthermore, the manual approach with predefined areas of interest^2^ (AOI) does not always meet the necessary standard for reliable analysis (Kredel et al., 2017). Provided that similar differences in gaze patterns, as found *in situ*, can be reproduced under controlled laboratory settings, the laboratory is preferable, according to Williams (2016), in order to isolate and better understand the basic mechanisms. In the present study, we try to minimize the aforementioned problem, by using a context similar to esport. In esport a participant seated in front of a stationary screen, a setting similar to a computerized laboratory setting used in this study. Thus, it is possible to eliminate some of the problems connected to *in situ* recording of data.

It can be argued that a first-person shooter setting in esport has some similarities with, for instance, clay-pigeon shooting or skeet, in which the goal is to hit a target that suddenly pops up and moves fast. That is, you have to detect the target, aim and shoot in a very limited time frame. The area where the target appears is limited and the shooter is stationary. The dissimilarities are of course the “gun” and the muscular activation needed to aim and to execute a shot. As shown above, the onset and duration of the QE is predictive for the shooting performance (i.e., Causer et al., 2010) in various shooting contexts.

The overall goal of this article is to investigate whether the QE can be replicated in a context similar to esport setting, where participants use the mouse to “shoot” targets appearing on a computer screen. This includes directing, as quickly and accurately as possible, the mouse cursor to the location of the target and “shooting” (clicking on) it. The QE will be considered within and between participants. First, we will address the question of how task performance is associated with QE parameters; are “hits” generally associated with a longer a QE duration and a shorter QE onset? Second, we analyze whether high-performing participants show a more distinct QE behavior compared to low-performing participants. Furthermore, by mimicking a challenging or critical moment during game play, we want to manipulate the player’s cognitive load. This is implemented by comparing a standard “shooting” task with a reverse-Stroop task. Finally, we utilize a state-of-the-art remote eye tracker, the Tobii Pro Spectrum, which provides data with higher sampling rates, accuracy, and precision in comparison to the data collected in previous studies of QE. The higher data quality offered by this eye tracker significantly increases the likelihood that participants’ “true” oculomotor fixations are measured, thus providing a more valid analysis of QE behavior. This, in combination with the knowledge of the exact locations of the targets on the computer screen, allow us to explore more elaborate analyses of QE parameters.

## Materials and Methods

### Participants

Twenty-three male university students between the ages of 18 and 30 with normal or corrected-to-normal vision volunteered to take part in the study. No data on dexterity were collected and the participants were free to choose which hand to wield the mouse with. All 23 participants were recruited on the campus area of Lund University. To estimate how familiar participants were with FPS games, they were asked if they played FPS games more than 5 h a month (yes/no). Eleven participants reported playing five or more hours of FPS games per month, whereas the remaining 12 said they played less than 5 h of FPS games per month. Since many of the participants felt it was difficult to quantify their gaming experience this way, we did not take gaming experience into account in the analysis. In accordance with Swedish law regulating research projects involving humans (SFS, 2003:460), no application for ethical approval was needed.

### Apparatus, Stimuli, and Procedure

Gaze data were recorded at 300 Hz using the Tobii Pro Spectrum (firmware 1.7.8) eye tracker and the Titta toolbox (Niehorster et al., 2020). Mouse cursor movement was recorded at 60 Hz using PsychoPy (Peirce, 2007, 2009). To synchronize mouse and gaze data, mouse position was sampled during a callback function that was called every time a gaze sample was generated by the eye tracker. The distance between the screen and the participant’s eyes was 63 cm, and their heads were stabilized with a custom built chin-and-forehead rest. The test, aiming to simulate an FPS esport environment, was created in PsychoPy (Peirce, 2007, 2009). The test consisted of two blocks with 64 “shots” (mouse clicks) in each block and a 30-s pause between the blocks. Both blocks were preceded by a practice run (8 shots). In the first block, a cross appeared in the middle of the screen, and the participants were instructed to click on the cross. Once clicked, a circular target (0.65° in diameter) appeared on the screen after a delay randomly chosen from the interval 500–2000 ms, and stayed on the screen for 860 ms. The target appeared randomly in one of eight, evenly spaced directions (0, 45, 90, 135, 180, 225, 270, 315 deg) on a perimeter 12° from the cross in the center of the screen. The task was to, as quickly and precisely as possible, “shoot” the target by moving the cursor and clicking the mouse. If the target was hit, it disappeared; if it was missed or if 860 ms elapsed without any click on the target, it disappeared and the cross reappeared in the center of the screen, prompting the participant to initiate the next shot.

In the second block, a reverse-Stroop task was introduced in order to increase the cognitive load, concordant with previous research on cognitive load and the QE (Wood et al., 2016). After the participant clicked on the cross in the center, either the word “blue” or “red” written in either blue or red color was shown in the center of the screen for 1000 ms. After another 500 ms, two targets appeared, one blue and one red. The participants were instructed to ignore the color of the word, and instead shoot the target with the color corresponding to the word itself. Since this task was assumed to be a bit more difficult, the time that the participants were given was increased by 50 ms from 860 ms (subtest one 1) to 910 ms. Gaze behavior and cursor movement were recorded continuously throughout the tests. The first block was designed to work as a baseline in comparison to the second block in terms of cognitive load.

A five-point calibration followed by a four-point validation of the calibration accuracy was performed for each participant prior to each recording. A recording was allowed to start only if the average accuracy across the validation targets was below one degree, and the precision estimated by the root-mean-square (RMS) of inter-sample distances was less than 0.1 degree, for both eyes. All participants met these criteria.

### Data Analysis

Quiet eye (QE) *onset* was the time from the appearance of the target until the onset of the last fixation within three degrees from the target before the mouse click. *QE duration* was operationalized as the time from *QE onset* until the end of the fixation that started at QE onset. *Mouse movement latency* was defined as the time from the appearance of the target to initiation of mouse movement, and was considered to happen when the mouse position was further than one degree away from the center of the screen, where the mouse was located at every trial onset. *Time to mouse click* was defined as the time from trial onset to the time when the “shot” was fired (the mouse was clicked).

Performance was quantified in two ways: first, as a binary hit/miss variable, and second, as a continuous variable operationalized as the distance between the center of the target and the position of the mouse cursor at the time of the mouse click. A “hit” was considered to happen if the mouse click was located on the target; otherwise the target was “missed.” Trials where the mouse click occurred after the offset of the target were excluded from the analysis.

Fixations were identified using the I2MC algorithm (Hessels et al., 2017). Settings related to screen distance, screen size and recording frequency were adjusted according to our particular setup. For the remaining parameters, default settings were used. The minimum fixation duration was set to 100 ms. Only trials where participants clicked the mouse before target offset, and at least one fixation was located within a three-degree radius from the target prior to mouse click were considered. Moreover, QE onsets and mouse movement latencies shorter than 100 ms were excluded.

Data were analyzed with Python 3.6 and R (v. 1.0.2) using the lme4 package (v. 1.1.21). When using (generalized) linear mixed-effects models, participants were treated as random variables with random intercepts.

## Results

In total, 2644 trials were analyzed (23 participants each performing two tasks with 64 trials each). After excluding trials, as explained in the “Data analysis” section, 2311 trials remained. These were used in the remainder of the analysis.

**FIGURE 1:**
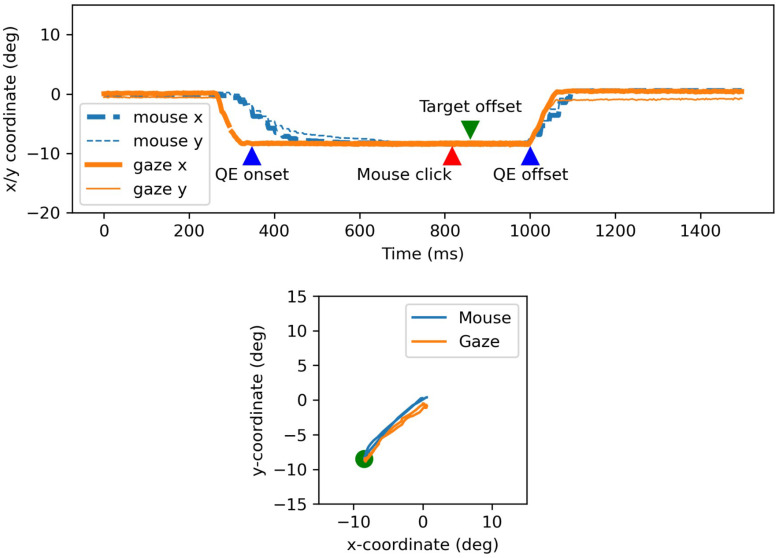
Example of mouse and gaze data collected from one trial over time (top) and space (bottom). The green dot in the bottom subfigure represents the target. Data from the left eye are shown.

An example of a trial is given in Figure 1, which shows how eye and mouse positions typically unfold over time (top row) and in space (bottom row). At trial onset, both mouse and gaze positions were located in the center of the screen. After about 250 ms (all trials: *M* = 230 ms, *SD* = 50 ms), a saccade was launched toward the target and shortly after, a mouse movement toward the target was initiated (all trials: *M* = 301 ms, *SD* = 55 ms). The QE fixation started directly after the initial saccade had landed on the target. The mouse was clicked before the disappearance of the target (860 ms), and the QE fixation continued for about another 140 ms.

### Mouse Results

**FIGURE 2:**
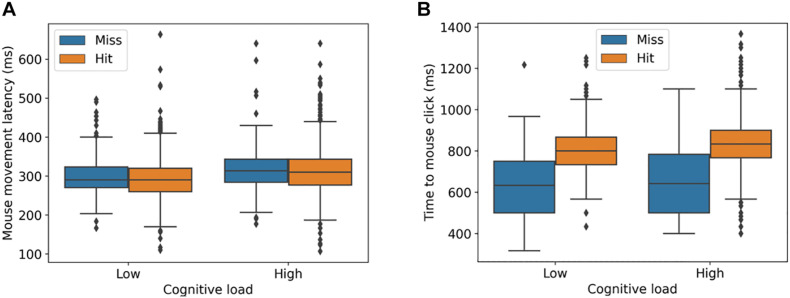
Mouse movement latency **(A)** and time to mouse click **(B)** for different levels of cognitive load. Misses and hits denote when the mouse click is on the target (hit) and outside of the target (miss).

Participants hit the targets in 88.1% (SD = 32.2) of the cases (low cognitive load: *M* = 88.8%, SD = 31.6%; high cognitive load: *M* = 87.6%, SD = 32.9%). There was no significant difference between the high and low cognitive load conditions, β = 0.10 (SE = 0.16), *z* = 0.68, *p* = 0.53. As the cognitive load increased (Figure 2), the mouse movement latency became significantly higher [Low: *M* = 295.2 ms, SD = 52.3 ms; High: *M* = 315.0 ms, SD = 57.8 ms; β = 17.2 (SE = 5.7), *t* = 2.9, *p* < 0.01] and the time to mouse click became significantly longer [Low: *M* = 785.9 ms, SD = 122.9 ms; High: *M* = 815.0 ms, SD = 138.5 ms; β = 42.9 (SE = 11.7), *t* = 3.6, *p* < 0.01]. Moreover, the time to mouse click was significantly longer for hits than for misses [Miss: *M* = 645.8 ms, SD = 164.1 ms; Hit: *M* = 821.5 ms, SD = 111.7 ms; β = 60.3 (SE = 10.1), *t* = 5.9, *p* < 0.01]. For mouse movement latency, there was no significant difference between hits and misses [Miss: *M* = 312.6 ms, SD = 62.1 ms; Hit: *M* = 304.4 ms, SD = 55.1 ms; β = –6.0 (SE = 4.98.1), *t* = –1.2, *p* = 0.23]. None of the participants clicked on (“hit”) the wrong target in the high cognitive load (Stroop) block. The performance between the left (180 deg), right (0 deg), top (90 deg), and bottom (270 deg) directions ranged between 83% (left) to 91% (down) across both blocks. We fit a linear mixed effect model predicting mouse hit accuracy (distance from mouse click to bullseye) from target position (left, right, up, down), using participants as random effects. Pairwise differences in accuracy between the target positions were tested using the emmeans package (v. 1.4.1). None of the pairwise differences was significant (all *p*-values > 0.33).

### Eye Movement Results

**FIGURE 3:**
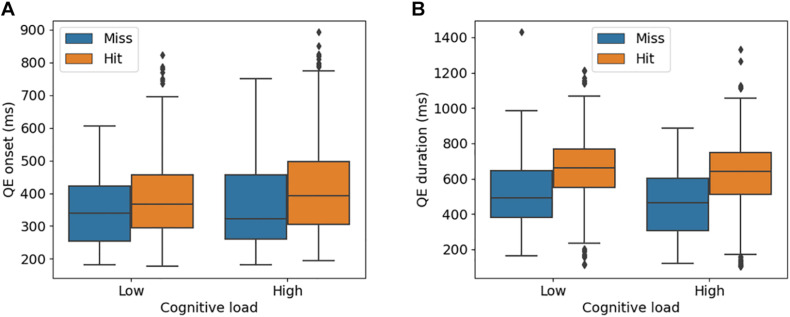
Quiet Eye (QE) onset **(A)** and duration **(B)** for different levels of cognitive load. Misses and hits denote when the mouse click is on the target (hit) and outside of the target (miss).

There was a significant effect of cognitive load on QE onset (Figure 3A), where a higher cognitive load was associated with later QE onsets [Low: *M* = 379.8 ms, SD = 119.1 ms; High: *M* = 417.4 ms, SD = 5 131.6 ms; β = 46.1 (SE = 14.4), *t* = 3.18, *p* < 0.01]. However, there was no significant difference between hits and misses [Miss: *M* = 365.3 ms, SD = 130.4 ms; Hit: *M* = 403.6 ms, SD = 125.9 ms; β = 8.9 (SE = 12.3), *t* = 0.72, *p* = 0.46]. For QE duration, there was no significant difference between low and high cognitive loads [Low: *M* = 621.0 ms, SD = 203.3 ms; High: *M* = 589.3 ms, SD = 202.7 ms; β = –37.1 (SE = 22.0), *t* = –1.68, *p* = 0.09], but hits had significantly longer durations in comparison with misses [Miss: *M* = 464.7 ms, SD = 205.6 ms; Hit: *M* = 623.6 ms, SD = 195.8 ms; β = 40.5 (SE = 18.9), *t* = 2.14, *p* = 0.03].

The alternative way of analyzing the data, and in our opinion more elaborate, is to follow the suggestion by Williams (2016), where performance is regarded as a continuous variable. The center of the target is considered “bullseye” and the distance between the “bullseye” and the actual mouse click defines the accuracy of the mouse click. Also, since QE behavior may be influenced not only by hit accuracy, but also the time until the ‘shot’ is fired (hit speed), we model QE duration and QE onset as a function of both speed and accuracy. For QE onset, there was a main effect of speed [β = –2.82 (SE = 0.35), *t* = –8.04, *p* < 0.001), but not accuracy [β = –6.38 (SE = 4.40), *t* = –1.44, *p* = 0.15]. The main effect of speed means that the longer it takes to click the mouse, the earlier the QE onset is. For QE duration, both speed [β = 1.70 (SE = 0.53), *t* = 3.17, *p* = 0.002], and accuracy [β = –19.1 (SE = 6.84), *t* = –2.79, *p* = 0.005] were significant; later and more accurate mouse clicks leads to longer QE durations. There were no significant interactions, neither for QE onset nor QE duration (*p* > 0.3 in both cases).

**FIGURE 4:**
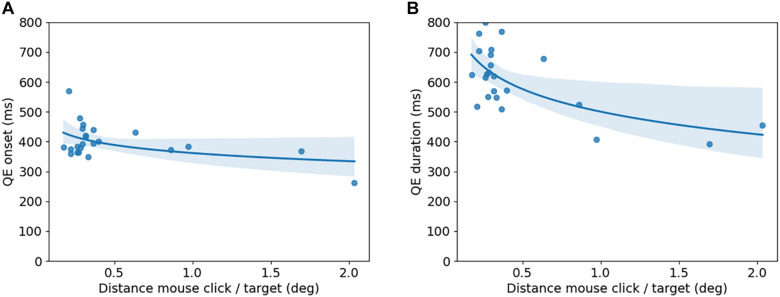
QE onset **(A)** and duration **(B)** as a function of distance to the target at mouse click. Each dot represents one participant. The line shows a logarithmic fit to the data and the shaded area represents a 95% confidence interval. Note that mouse clicks both in (hits) and outside (misses) the target are included.

To assess whether high-performing participants showed a more distinct QE behavior, the relationship between the hit accuracy QE onset and QE duration is plotted in Figures 4A,B, respectively, where each dot represents one participant. There was a significant Spearman rank-order correlation between both D and QE onset (*r* = –0.46, *p* = 0.03), and D and QE duration (*r* = –0.75, *p* < 0.01), indicating a monotonicity of the relationship between the variables.

In the definition by Vickers (1996), a fixation is only classified as a QE fixation if it falls within three degrees of the target. Changing this value to two and four degrees changed the results only marginally. For example, the correlations in Figure 4 changed from –0.75 (QE duration) and –0.46 (QE onset) when using a threshold of 3 to –0.71 (QE duration) and –0.39 (QE onset) for a threshold of 2, and –0.78 (QE duration) and –0.47 (QE onset) for a threshold of 4.

## Discussion

In this study, we have used a computerized precision task in a setup similar to those found in first-person shooter (FPS) e-games, in order to investigate whether findings about the “quiet eye” (QE), traditionally found in other sports-related tasks (e.g., shotgun shooting: Causer et al., 2010; basketball: Rienhoff et al., 2013; biathlon shooting: Vickers and Williams, 2007; dart-throwing: Nibbeling et al., 2012) generalize to a computer setup. Participants were asked to “hit” (click on) briefly appearing targets on a computer screen as quickly and accurately as they could in low and high cognitive load conditions. Most importantly, we could replicate the QE, with longer QE durations for “hits” than for “misses.” Consequently, and consistent with previous work, a prolonged QE could be seen as an indicator of successful performance. However, unlike previous studies, we did not find a significant difference between hits and misses with respect to QE onset. Similar results were found when, instead of a binary hit-miss variable, analyzing performance as the relationship between the QE duration (or QE onset) and the distance between the mouse click and the target center (“bullseye”). Also considering the time it took until the mouse was clicked (speed), it became clear that the QE duration was influenced by both the speed and accuracy at which the target was clicked; longer QE durations were associated with both more accurate and later mouse clicks, but shorter QE onsets only with later mouse clicks.

Comparing QE parameters between participants, significant negative correlations were found between hit accuracy and QE duration/onset. Figure 4B showed, as expected, a prolonged QE duration for more accurate hits. Figure 4A showed, unexpectedly, that less accurate hits were accompanied by earlier QE onsets. This pattern is contrary to what has previously been reported in the QE literature. The reason for this is not clear and needs to be further studied.

One interpretation of the results in Figure 4 is that different participants may have optimized on different behaviors when conducting the task. The task instruction to “as quickly and accurately as possible, ‘shoot’ the target” allows participants to optimize on either speed or accuracy. It is conceivable that participants with poorer accuracy (represented by dots in Figure 4B) tried to perform the task as quickly as possible while compromising accuracy. At the other extreme (represented by dots in Figure 4A), participants tried to use most of the available time on aiming and clicking as accurately as possible, leading to long QE duration on the target. Indeed there was a strong correlation between speed and accuracy, with a Pearson correlation of *r* = –0.83 (*p* < 0.001).

We argue that it is more informative to analyze performance as a continuous variable rather than as a dichotomous variable. The reason for this is that in many settings, performance is a question of absolute accuracy rather than “all or nothing.” This is obvious in sports where the target consists of a graded scale, which is the case in, e.g., target shooting, where a “bullseye” or a “10” reflect a perfect hit, and lower values reflect less-accurate hits. Furthermore, we argue that this is also a more suitable form of analysis in other sports such as soccer, in which there is no static “bullseye.” In such cases, you have a designated target (the goal), but in order to score a goal you have to get the ball past the goalkeeper. In one situation, this could mean targeting the area to the lower right, while in another situation it means getting the ball just below the bar to the left. In other words, the “bullseye” moves within the designated target (the goal). Measuring performance as a continuous variable makes it possible to get a more elaborate measure of the precision or skill in a particular task in any given situation, rather than just comparing it dichotomously. In biathlon, for instance, two athletes can have the same numbers of hits, but the hits can vary greatly in proximity to the bullseye. The same is probably true in other settings outside of sports. One example of where detailed analysis of gaze behavior and target hit accuracy could be useful is in the evaluation of police officers’ ability to use various forms of countermeasures.

Besides the fact that our study is conducted with a computer setup with a screen and mouse, there are some important differences between many previous QE- studies and the current study First, in previous studies on, e.g., golf and basketball, the tasks are mostly self-paced and performed without a strict time pressure. In these tasks, accuracy is more important than speed, and it is difficult to adjust the movement of the hands/golf club during the actual movement execution. Here, at least for quick participants, there was a chance to correct the initial landing position of the mouse to obtain a more accurate hit.

In this study, we used a screen-based test similar to an FPS action video game. There are obvious technical advantages to using eye trackers in this context, since the experimental setting coincides with the setting used in esport: a relatively static participant in front of the computer screen. There are of course, other differences between the simple and static task used in this experiment and the very complex environment of a modern AVGs (multiple targets, moving targets, stress inducing situations, etc.), aspects that we intend to address in coming studies. Unlike the majority of previous studies investigating the QE using mobile, head-worn eye trackers, we have presented stimuli on a computer screen and used a high-end eye tracker (the Tobii Pro Spectrum) to collect eye movement data. Consequently, the data we have analyzed in this paper typically have higher sample rates, accuracy and precision in comparison with data in the older studies. This has several important implications. First, the original QE definition says that the eye gaze needs to be within three degrees of the target (Vickers, 1996). This probably reflects the (in)-accuracy of mobile eye trackers at that time. The accuracy of our data was below one degree for all participants, meaning that Vickers’s threshold of three degrees is probably unnecessarily high. However, changing this threshold to two or four degrees did not change our results significantly. Second, and perhaps even more important, the higher sample rates and precision of the data make it easier to detect smaller saccades. Thus, the exact same oculomotor fixation of the eye may end up having a different (QE) duration when computed from data recorded with different eye trackers. More generally, the duration of the QE depends critically on a number of factors, including (1) the sample rate and precision of the data, (2) the particular algorithm used to detect it, and (3) the theoretical criteria that are used to separate larger voluntary saccades from microsaccades (*cf.* Poletti and Rucci, 2016). Consequently, the definition of the QE is intimately connected to data quality, data processing, and definitions of what fixations and saccades are (for a more detailed discussion on this topic, see Hessels et al., 2018).

From the first esport event held in 1972 at Stanford University, mainly for local students, esport has grown to a multi-billion-dollar industry, where the number of spectators of the most popular events, such as the world championship final of *League of Legends*, equals or surpasses “traditional” events such as Super Bowl, and the prognosis says that popularity is still rapidly increasing (NewZoo.com, 2020). The same can be said about the hardware and the programming behind the interface of the games, where the technical performance of computers powering the games has increased by several million percent over the two last decades. Research in the area of esports, however, has not experienced a similar increase. While it has grown from being practically non-existent in the early 2000s to now being spread across several academic fields (for a review of esport research, see Dale et al., 2020; Reitman et al., 2020), the number of published articles remains surprisingly low. This will probably change in the future, however. The results presented in this paper show that QE may also be beneficial in an esport context, and that esport practitioners should therefore be aware of its existence.

A difference in performance related to cognitive load has typically been found in various types of tasks (i.e., Wilson et al., 2015; Vickers, 2016). In this paper, higher levels of cognitive load led to a generally slower performance, indicated by a slower mouse movement initiation, a later mouse click on the target, and a later onset of the QE fixation. This likely reflects the additional time required to process both the possible mismatch between the color of the text and the meaning of the text, and visually discriminate between two possible targets instead of one. However, cognitive load did not significantly influence the duration of the QE. One particular challenge associated with manipulating cognitive load is its dynamic nature (e.g., Hanslmayr et al., 2008), and it is uncertain whether the Stroop manipulation used in this paper actually led to a higher degree of cognitive load at the moment of the critical event, the mouse click, and would thus influence QE duration. In order to further test the impact of cognitive load other types of manipulations than Stroop need to be investigated.

The participant sample consisted of university students who had not been pre-screened about their gaming habits. About half of the participants reported playing less than 5 h of FPS games per month, and the other half played more or equal to 5 h per month. Thus, it is unclear how the results in this paper would generalize to those from professional gamers, routinely playing FPS games several hours a week.

## Conclusion

We have replicated the “quiet eye” (QE) effect, found in a variety of sports, using a high-end eye tracker in a computerized task similar to FPS computer games. QE, the duration of the last fixation before a motor action (in our case clicking the mouse on a target), predicts the performance outcome. We argue that a more elaborate way of analyzing the data is to treat them as continuous variables, that is, to look at the distance between the center of the target and the actual hit, rather than dichotomously looking at “hits” versus “misses.” Both QE duration and QE onset were significantly negatively correlated with the distance between the mouse click on the target and the center of the target (“bullseye”). This latter correlation, however, was unexpected and needs to be further investigated.

## Data Availability Statement

The raw data supporting the conclusions of this article will be made available by the authors, without undue reservation.

## Ethics Statement

Ethical review and approval was not required for the study on human participants in accordance with the local legislation and institutional requirements. The patients/participants provided their written informed consent to participate in this study.

## Author Contributions

MD, MT, AH, and MN contributed to conception and design of the study and wrote sections of the manuscript. MN performed the statistical analysis. MD wrote the first draft of the manuscript. All authors contributed to manuscript revision, read, and approved the submitted version.

## Conflict of Interest

The authors declare that the research was conducted in the absence of any commercial or financial relationships that could be construed as a potential conflict of interest.

## Publisher’s Note

All claims expressed in this article are solely those of the authors and do not necessarily represent those of their affiliated organizations, or those of the publisher, the editors and the reviewers. Any product that may be evaluated in this article, or claim that may be made by its manufacturer, is not guaranteed or endorsed by the publisher.

## Footnotes

1 The “hot hand” is the belief that an athlete’s performance during a particular period of, i.e., a basketball match is significantly better than the average performance of the athlete (Gilovich et al., 1985).

2 For a discussion on the problem with AOI, see Hessels et al. (2016).

## References

[B1] Bediou B. Adams D. M. Mayer R. E. Tipton E. Green C. S. Bavelier D. (2018). Metaanalysis of action video game impact on perceptual, attentional, and cognitive skills. *Psychol. Bull.* 144 77–110. 29172564 10.1037/bul0000130

[B2] Causer J. Bennett S. J. Holmes P. S. Janelle C. M. Williams A. M. (2010). Quiet eye duration and gun motion in elite shotgun shooting. *Med. Sci. Sports Exerc.* 42 1599–1608. 10.1249/MSS.0b013e3181d1b05 20139787

[B3] Causer J. Harvey A. Snelgrove R. Arsenault G. Vickers J. N. (2014). Quiet eye training improves surgical knot tying more than traditional technical training: A randomized controlled study. *Am. J. Surg.* 208 171–177. 10.1016/j.amjsurg.2013.12.042 24881015

[B4] Choi G. Kim M. (2015). Eye-movement pattern by playing experience in combat system of FPS game. *Adv. Sci. Technol. Lett.* 96 52–56.

[B5] Collewijn H. Kowler E. (2008). The significance of microsaccades for vision and oculomotor control. *J. Vis.* 8:20. 10.1167/8.14.20 PMC3522523 19146321

[B6] Corbetta M. Patel G. Shulman G. L. (2008). The reorienting system of the human brain: From environment to theory of mind. *Neuron* 58 306–324. 10.1016/j.neuron.2008.04.017 18466742 PMC2441869

[B7] Corbetta M. Shulman G. L. (2002). Control of goal-directed and stimulus-driven attention in the brain. *Nat. Rev. Neurosci.* 3 201–215. 10.1038/nrn755 11994752

[B8] Dale G. Joessel A. Bavelier D. Green C. S. (2020). A new look at the cognitive neuroscience of video game play. *Ann. N. Y. Acad. Sci.* 1464 192–203. 10.1111/nyas.14295 31943260

[B9] Gilovich T. Vallone R. Tversky A. (1985). The hot hand in basketball: On the misperception of random sequences. *Cogn. Psychol.* 17 295–314.

[B10] Gonzalez G. C. Causer J. Miall R. C. Grey M. J. Humphreys G. Williams A. M. (2017). Identifying the causal mechanisms of the quiet eye. *Eur. J. Sport Sci.* 17 74–84. 10.1080/17461391.2015.1075595 26356536

[B11] Gorbet D. J. Sergio L. E. (2018). Move faster, think later: women who play action video games have quicker visually guided responses with later onset visuomotor-related brain activity. *PLoS One* 13:e0189110. 10.1371/journal.pone.0189110 29364891 PMC5783344

[B12] Hamari J. Sjöblom M. (2017). What is eSports and why do people watch it? *Internet. Res.* 27 211–232. 10.1108/IntR-04-2016-0085

[B13] Hanslmayr S. Pastotter B. Bauml K. H. Gruber S. Wimber M. Klimesch W. (2008). The electrophysiological dynamics of interference during the stroop task. *J. Cogn. Neurosci.* 20, 215–225. 18275330 10.1162/jocn.2008.20020

[B14] Hessels R. S. Kemner C. van den Boomen C. Hooge I. T. C. (2016). The area-of-interest problem in eyetracking research: A noise-robust solution for face and sparse stimuli. *Behav. Res. Methods* 48 1694–1712. 10.3758/s13428-015-0676-y 26563395 PMC5101255

[B15] Hessels R. S. Niehorster D. C. Kemner C. Hooge I. T. C. (2017). Noise-robust fixation detection in eye-movement data – Identification by 2-means clustering (I2MC). *Behav. Res. Methods* 49 1802–1823. 10.3758/s13428-016-0822-1 27800582 PMC5628191

[B16] Hessels R. S. Niehorster D. C. Nyström M. Andersson R. Hooge I. T. (2018). Is the eye-movement field confused about fixations and saccades? A survey among 124 researchers. *R. Soc. Open Sci.* 5:180502. 10.1098/rsos.180502 PMC6124022 30225041

[B17] Hüttermann S. Noel Memmert D. (2018). Eye tracking in high-performance sports: evaluation of its application in expert athletes. *Int. J. Comput. Sci. Sport* 17, 182–203. 10.2478/ijcss-2018-0011

[B18] Klostermann A. Hossner E.-J. (2018). The quiet eye and motor expertise: explaining the “efficiency paradox”. *Front. Psychol.* 9:104. 10.3389/fpsyg.2018.00104 29472882 PMC5809435

[B19] Klostermann A. Kredel R. Hossner E.-J. (2014). On the interaction of attentional focus and gaze: The quiet eye inhibits focusrelated performance decrements. *J. Sport Exerc. Psychol.* 36 392–400. 10.1123/jsep.2013-0273 25226608

[B20] Klostermann A. Moeinirad S. (2020). Fewer fixations of longer duration? Expert gaze behavior revisited. *German J. Exerc. Sport Res.* 50 146–161. 10.1007/s12662-019-00616-y

[B21] Koposov D. Semenova M. Somov A. Lange A. Stepanov A. Burnaev E. (2020). *Analysis of the Reaction Time of eSports Players through the Gaze Tracking and Personality Trait.* Piscataway: IEEE, 1560–1565. 10.1109/ISIE45063.2020.9152422

[B22] Kredel R. Vater C. Klostermann A. Hossner E.-J. (2017). Eye-tracking technology and the dynamics of natural gaze behaviour in sports: A systematic review of 40 years of research. *Front. Psychol.* 8:1845. 10.3389/fpsyg.2017.01845 29089918 PMC5651090

[B23] Lebeau J. C. Liu S. Sáenz-Moncaleano C. Sanduvete-Chaves S. Chacón-Moscoso S. Becker B. J. (2016). Quiet eye and performance in sport: a meta-analysis. *J. Sport Exerc. Psychol.* 38 441–457. 27633956 10.1123/jsep.2015-0123

[B24] Li R. Polat U. Makous W. Bavelier D. (2010). Enhancing the contrast sensitivity function through action video game training. *Nat. Neurosci.* 12 549–551. 10.1038/nn.2296 PMC2921999 19330003

[B25] Miles C. A. L. Wood G. Vine S. J. Vickers J. N. Wilson M. R. (2015). Quiet eye training facilitates visuomotor coordination in children with developmental coordination disorder. *Res. Dev. Disabil.* 40 31–41. 25721344 10.1016/j.ridd.2015.01.005

[B26] Moore L. J. Vine S. J. Wilson M. R. Freeman P. (2012). The effect of challenge and threat states on performance: An examination of potential mechanisms. *Psychophysiology* 49 1417–1425. 10.1111/j.1469-8986.2012.01449.x 22913339 PMC3677799

[B27] Nibbeling N. Oudejans R. R. Daanen H. A. (2012). Effects of anxiety, a cognitive secondary task, and expertise on gaze behavior and performance in a far aiming task. *Psychol. Sport Exerc.* 13, 427–435.

[B28] Niehorster D. C. Andersson R. Nyström M. (2020). Titta: A toolbox for creating PsychToolbox and Psychopy experiments with Tobii eye trackers. *Behav. Res.* 52 1970–1979. 10.3758/s13428-020-01358-8 32128697 PMC7575480

[B29] Pardina-Torner H. Carbonell X. Castejón M. (2019). A comparative analysis of the processing speed between video game players and non-players. *Aloma* 37 13–20.

[B30] Peirce J. W. (2007). PsychoPy – Psychophysics software in Python. *J. Neurosci. Methods* 162, 8–13. 10.1016/j.jneumeth.2006.11.017 17254636 PMC2018741

[B31] Peirce J. W. (2009). Generating stimuli for neuroscience using PsychoPy. *Front. Neuro.* 2, 1–8. 10.3389/neuro.11.010.2008 19198666 PMC2636899

[B32] Piras A. Vickers J. N. (2011). The effect of fixation transitions on quiet eye duration and performance in the soccer penalty kick: Instep versus inside kicks. *Cogn. Process.* 12 245–255. 10.1007/s10339-011-0406-z 21544570

[B33] Poletti M. Rucci M. (2016). A compact field guide to the study of microsaccades: Challenges and functions. *Vis. Res.* 118 83–97. 25689315 10.1016/j.visres.2015.01.018 PMC4537412

[B34] Powers K. L. Brooks P. J. Aldrich N. J. Palladino M. A. Alfieri L. (2013). Effects of video-game play on information processing: a meta analytic investigation. *Psychon. Bull. Rev.* 20 1055–1079. 23519430 10.3758/s13423-013-0418-z

[B35] Reitman J. G. Anderson-Coto M. J. Wu M. Lee J. S. Steinkuehler C. (2020). Esports research: A literature review. *Games Cult.* 15:155541201984089. 10.1177/1555412019840892

[B36] Rienhoff R. Hopwood M. J. Fischer L. Strauss B. Baker J. Schorer J. (2013). Transfer of motor and perceptual skills from basketball to darts. *Front. Psychol.* 4:593. 10.3389/fpsyg.2013.00593 24062703 PMC3771373

[B37] Rienhoff R. Tirp J. Strauss B. Baker J. Schorer J. (2016). The “quiet eye” and motor performance: a systematic review based on Newell’s constraints-led model. *Sports Med.* 46, 589–603. 10.1007/s40279-015-0442-4 26712511

[B38] SFS (2003). *460 Lagen om etikprövning av forskning som avser människor.* Stockholm: Riksdagen.

[B39] Stroop J. R. (1935). Studies of interference in serial verbal reactions. *J. Exp. Psychol*. 18 643–662.

[B40] Vickers J. N. (1996). Visual control when aiming at a far target. *J. Exp. Psychol. Hum. Percept. Perform.* 22 342–354. 8934848 10.1037//0096-1523.22.2.342

[B41] Vickers J. N. (2009). Advances in coupling perception and action: The quiet eye as a bidirectional link between gaze, attention, and action. *Prog. Brain Res.* 174 279–288. 10.1016/S0079-6123(09)01322-3 19477346

[B42] Vickers J. N. (2016). Origins and current issues in quiet eye research. *Curr. Issues Sport Sci.* 1:101. 10.15203/CISS_2016.101

[B43] Vickers J. N. Lewinski W. (2012). Performing under pressure: Gaze control, decision making and shooting performance of elite and rookie police officers. *Hum. Mov. Sci.* 31 101–117. 10.1016/j.humov.2011.04.004 21807433

[B44] Vickers J. N. Williams A. M. (2007). Performing under pressure: The effects of physiological arousal, cognitive anxiety, and gaze control in biathlon. *J. Mot. Behav.* 39 381–394. 10.3200/JMBR.39.5.381-394 17827115

[B45] Wang P. Liu H.-H. Zhu X.-T. Meng T. Li H. J. Zuo X. N. (2016). Action video game training for healthy adults: a meta-analytic study. *Front. Psychol.* 7:907. 10.3389/fpsyg.2016.00907 27378996 PMC4911405

[B46] Williams A. M. (2016). Quiet eye vs. noisy brain: The eye like the brain is always active – comment on Vickers. *Curr. Issues Sport Sci.* 1:116. 10.15203/CISS_2016.116

[B47] Wilson M. Causer J. Vickers J. (2015). “Aiming for excellence: The quiet eye as a characteristic of expertise,” in *Handbook of Sport Expertise*, eds Baker J. Farrow D. (London: Routledge/Taylor and Francis), 22–37.

[B48] Wood G. Vine S. Wilson M. (2016). Working memory capacity, controlled attention and aiming performance under pressure. *Psychol. Res.* 80 510–517. 10.1007/s00426-015-0673-x 26021749

