# Supplementary material for: Quiet Eye and Computerized Precision Tasks in First-Person Shooter Perspective Esport Games
Source: Front Psychol. 2021 Nov 8;12:676591. doi: 10.3389/fpsyg.2021.676591 (PMC8606425; doi:10.3389/fpsyg.2021.676591)

Appendix

Table A1. Output of the generalized linear mixed-effect model predicting performance (hits/misses in mouse clicks) from task (low or high workload). Participants (pid) were used as random effects with random intercepts.


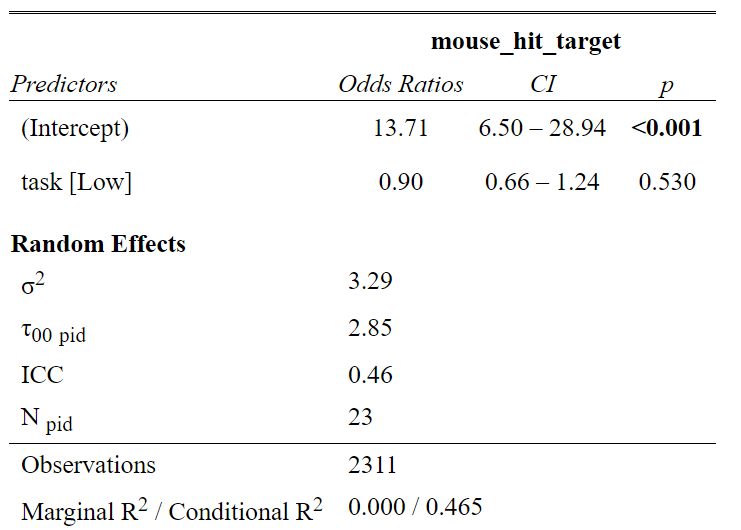


Table A2. Output of the linear mixed-effect model for mouse movement latency and time to mouse click. Independent variables are the task (low or high workload) and mouse performance (hit or miss target with a mouse click). Participants (pid) were used as random effects with random intercepts.


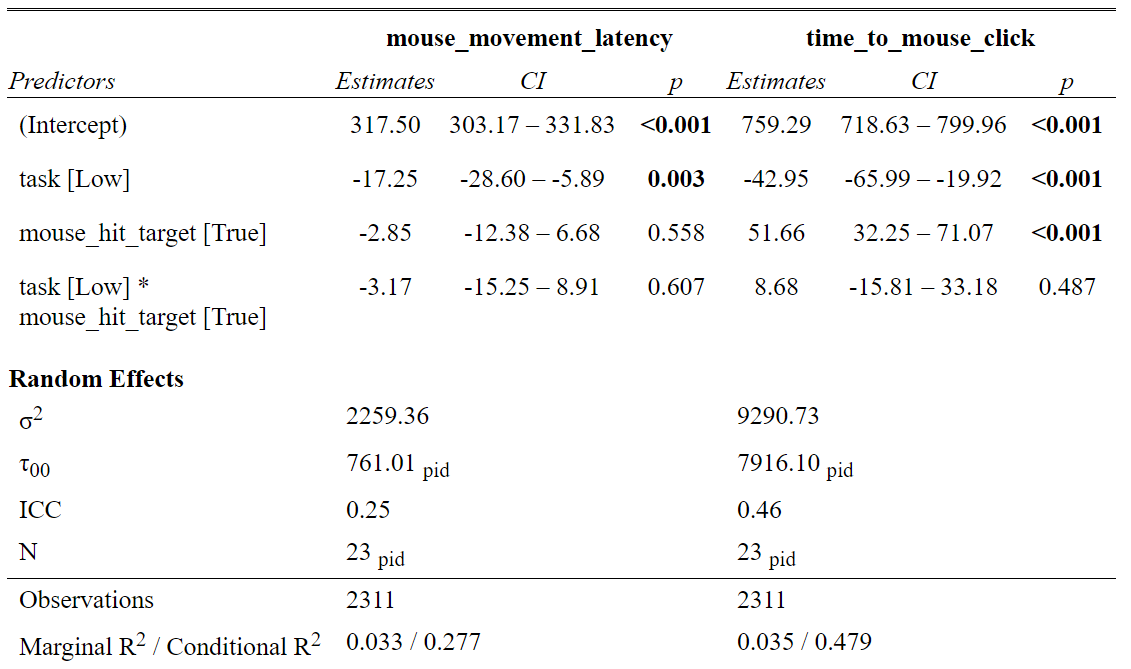


Table A3. Output of the linear mixed-effect model for quite eye (QE) onset and duration. Independent variables are the task (low or high workload) and mouse performance (hit or miss target with a mouse click). Participants (pid) were used as random effects with random intercepts.


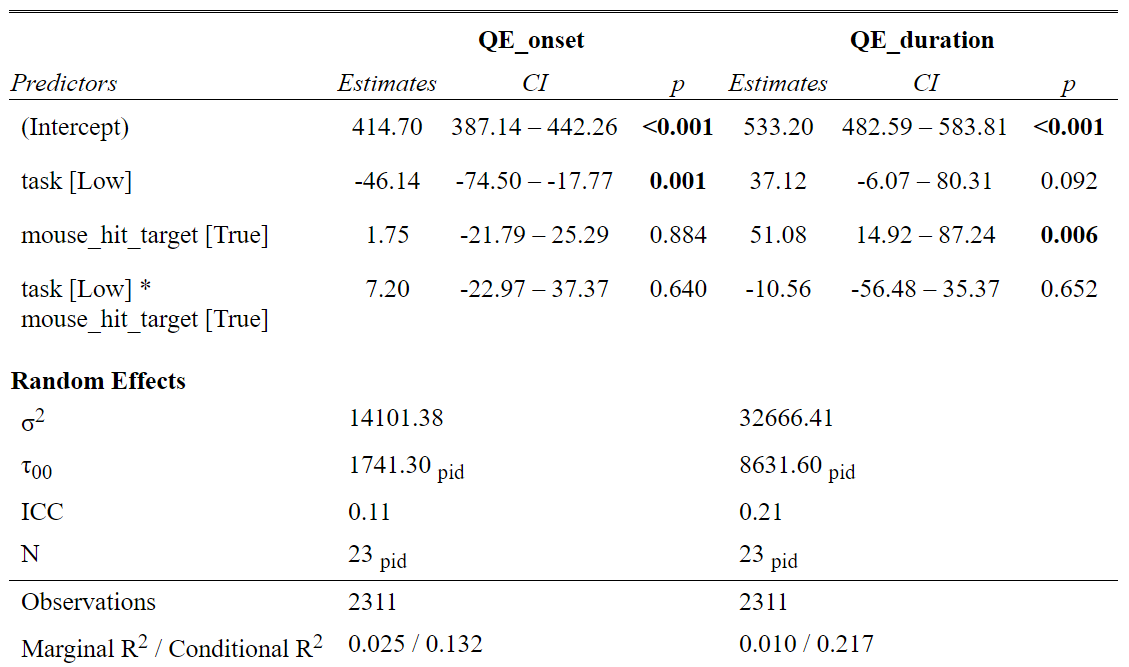

Supplement: Supplementary file 1 [file Table_1.DOCX]
